# Data on expression of genes involved in estrogen and progesterone action, inflammation and differentiation according to demographic, histopathological and clinical characteristics of endometrial cancer patients

**DOI:** 10.1016/j.dib.2017.04.050

**Published:** 2017-05-04

**Authors:** Maša Sinreih, Saša Štupar, Luka Čemažar, Ivan Verdenik, Snježana Frković Grazio, Špela Smrkolj, Tea Lanišnik Rižner

**Affiliations:** a Institute of Biochemistry, Faculty of Medicine, University of Ljubljana, Ljubljana, Slovenia; b Faculty of Medicine, University of Ljubljana, Ljubljana, Slovenia; c Division of Gynaecology and Obstetrics, University Medical Centre Ljubljana, Ljubljana, Slovenia; d Department of Pathology, Division of Gynaecology and Obstetrics, University Medical Centre Ljubljana, Ljubljana, Slovenia

**Keywords:** Gene expression, Endometrial cancer, Estrogens, Progesterone, Inflammation, Differentiation

## Abstract

Endometrial cancer is the sixth most common cancer in women worldwide. It is associated with aberrant actions of steroid hormones, estrogens and progesterone, but also with enhanced inflammation and reduced cellular differentiation. Here, we show data on demographic and histopathological characteristics of 51 patients with endometrial cancer, together with data on correlations between the expression of 38 genes involved in estrogen and progesterone actions, inflammation and differentiation, and demographic characteristics. We also show data on changes in gene expression of these 38 genes according to histopathological and clinical characteristics of these patients. This article includes data referenced in the manuscript entitled »STAR and AKR1B10 are down-regulated in high-grade endometrial cancer by Sinreih et al. (in press) [1].

**Specifications Table**

**Table:** 

Subject area	Biochemistry, Molecular biology
More specific subject area	Molecular endocrinology, Gynecological endocrinology
Type of data	Tables
How data was acquired	Clinical and histopathological data was obtained from the patients’ medical and histopathological records, respectively.
	The gene expression data obtained by quantitative real-time PCR was statistically analyzed.
Data format	Analyzed
Experimental factors	
Experimental features	Ratios for expression of 38 genes in samples of endometrial cancer *versus* adjacent control endometrium were calculated and this data was statistically analyzed.
Data source location	Ljubljana, Slovenia
Data accessibility	The statistically analyzed data is available within this article and the raw expression data may be provided upon request.

**Value of the data**

• Data on correlations between the expression ratios of these 38 genes and demographic characteristics may be helpful for explanation of different etiological factors identified in epidemiological studies.
• Data on changes in the expression ratios of these 38 genes according to histopathological and clinical data may lay foundation for further investigations of individual players of the individual pathophysiological processes.

## 1 Data

We provide data on demographic, histopathological and clinical characteristic of 51 endometrial cancer patients treated at the University Medical Centre Ljubljana, at the Division of Gynaecology and Obstetrics. Demographic (age, body mass, BMI, menopausal status, parity), histopathological and clinical data (histological type and grade of tumor, depth of myometrial invasion, presence of lymphovascular invasion, FIGO stage) (Table 1) together with data on statistical analysis of gene expression ratios (Table 2, Table 3, Table 4, Table 5, Table 6, Table 7, Table 8, Table 9, Table 10, Table 11) are included. The study was approved by the National Medical Ethics Committee of the Republic of Slovenia.

**Table 1:** Demographic, histopathological and clinical characteristics of the endometrial cancer patients.

**Sample**	**Age**	**Body mass**	**BMI**	**Age at last menstruation**	**Parity**	**FIGO stage**	**Histological grade (low/high)**	**Myometrial invasion (yes/no)**	**Myometrial invasion >1/2 (yes/no)**	**Lymphovascular invasion (yes/no)**
**1**	39	59	21.7	premenopausal	1	IB	high	yes	yes	yes
**2**	83	NA	NA	50	4	IB	high	yes	yes	no
**3**	41	130	46.1	premenopausal	1	IA	low	yes	no	no
**4**	53	79	28.3	50	1	IA	low	no	no	no
**5**	60	68	25.0	56	1	IB	low	yes	yes	yes
**6**	64	63	26.2	50	1	IV	low	yes	no	NA
**7**	73	95	34.1	45	1	IB	low	yes	yes	no
**8**	69	83	31.6	59	1	IA	low	yes	no	no
**9**	79	84	32.8	49	2	IB	low	yes	yes	no
**10**	74	75	28.6	50	1	IA	low	yes	no	no
**11**	76	83	32.4	56	1	IA	low	yes	no	no
**12**	53	70	27.3	premenopausal	3	IA	low	no	no	no
**13**	36	92	33.8	premenopausal	2	IA	low	no	no	no
**14**	45	55	20.0	premenopausal	1	IA	low	no	no	no
**15**	69	68	25.3	53		IB	low	yes	yes	yes
**16**	54	65	23.0	premenopausal	0	IA	high	NA	no	no
**17**	72	100	35.9	45	1	IA	low	NA	no	no
**18**	54	51	19.9	premenopausal	2	IA	low	no	no	no
**19**	69	82	30.1	65	0	IB	high	yes	yes	yes
**20**	77	85	NA	50	1	IB	high	yes	yes	no
**21**	57	104	38.2	56	2	IA	low		no	no
**22**	61	88	30.8	50	2	IA	low	no	no	no
**23**	78	69	NA	50	2	IA	low	yes	no	yes
**24**	63	75	31.6	55	3	IA	low	yes	no	yes
**25**	71	80	29.4	59	2	IA	high	yes	no	no
**26**	81	82	28.4	51	2	IA	low	NA	no	no
**27**	73	65	24.8	48	0	IB	high	yes	yes	yes
**28**	50	88	32.3	premenopausal	1	IIIA	low	yes	no	no
**29**	27	57	20.0	premenopausal	0	IA	high	yes	no	no
**30**	59	60	19.4	40	2	IB	high	yes	yes	yes
**31**	70	119	47.7	50	1	IA	low	yes	no	no
**32**	73	100	34.6	53	2	IA	low	yes	no	no
**33**	75	73	30.4	55	3	IA	low	yes	yes	yes
**34**	75	130	48.9	50	3	IA	low	yes	no	yes
**35**	50	86	NA	NA	1	IA	high	yes	no	yes
**36**	71	100	41.1	54	4	IA	low	yes	no	no
**37**	75	60	24.0	50	1	IIIC1	high	yes	yes	yes
**38**	55	95	38.1	54	4	IA	low	no	no	no
**39**	43	110	44.6	premenopausal	2	IA	low	no	no	no
**40**	68	87	34.9	53	2	IA	low	yes	no	no
**41**	83	90	33.1	55	2	IA	low	no	no	no
**42**	59	102	37.5	52	1	IA	low	no	no	no
**43**	66	93	34.6	57	1	IA	low	yes	no	no
**44**	66	67	25.5	52	2	IA	low	yes	no	no
**45**	80	59	28.1	50	1	IB	high	yes	yes	yes
**46**	72	67	27.5	50	3	IA	low	yes	no	no
**47**	44	79	29.0	premenopausal	2	IA	low	no	no	no
**48**	45	60	20.8	premenopausal	2	II	low	yes	no	no
**49**	72	80	29.4	58	2	IA	low	yes	no	no
**50**	55	97	NA	58	2	IB	high	yes	yes	NA
**51**	48	94	NA	premenopausal	2	IA	high	yes	no	no

NA, not available.

**Table 2:** Correlations between expression of genes involved in estrogen biosynthesis and action and demographic characteristics of endometrial cancer patients.

**Gene**	**Age**				**Body mass**				**BMI**				**Age at menopause**				**Parity**			
	**Rho**	***p***	**Adj.*p***	***N***	**Rho**	***P***	**Adj.**	***N***	**Rho**	***p***	**Adj. *p***	***N***	**Rho**	***p***	**Adj. *p***	***N***	**Rho**	***p***	**Adj.*p***	***N***
***AKR1C3***	-0.159	0.459	1.000	24	-0.029	0.895	0.895	23	-0.012	0.960	1.000	21	0.231	0.356	0.949	18	-0.473*	**0.022**	**0.352**	**23**
***CYP19A1***	-0.110	0.610	0.976	24	-0.108	0.623	0.906	23	-0.010	0.964	1.000	21	-0.032	0.899	1.000	18	0.129	0.557	1.000	23
***HSD17B2***	**0.355**	**0.017**	**0.272**	**45**	0.173	0.262	1.000	44	0.209	0.190	1.000	41	-0.360*	**0.043**	**0.688**	**32**	0.245	0.109	0.872	44
***HSD17B1***	0.000	0.998	0.998	29	-0.116	0.558	1.000	28	-0.179	0.381	1.000	26	0.107	0.652	0.869	20	0.059	0.767	1.000	28
***HSD17B4***	-0.097	0.654	0.872	24	-0.056	0.800	0.853	23	0.086	0.712	1.000	21	0.138	0.585	0.851	18	0.079	0.719	1.000	23
***HSD17B8***	0.018	0.934	1.000	24	-0.122	0.579	1.000	23	-0.203	0.378	1.000	21	0.028	0.913	1.000	18	-0.310	0.150	0.800	23
***HSD17B14***	-0.150	0.495	1.000	23	0.192	0.393	1.000	22	0.238	0.313	1.000	20	0.173	0.507	0.811	17	0.257	0.249	0.996	22
***HSD17B12***	0.115	0.593	1.000	24	0.090	0.683	0.911	23	-0.001	0.996	0.996	21	-0.278	0.264	1.000	18	-0.083	0.708	1.000	23
***SULT1E1***	0.192	0.255	1.000	37	0.127	0.461	1.000	36	0.122	0.499	1.000	33	-0.213	0.285	0.912	27	0.016	0.925	0.987	36
***STS***	0.109	0.573	1.000	29	0.063	0.751	0.858	28	0.062	0.763	1.000	26	0.171	0.472	0.839	20	-0.012	0.952	0.952	28
***SULT2A1***	-0.101	0.553	1.000	37	-0.088	0.610	0.976	36	-0.077	0.669	1.000	33	0.019	0.926	0.988	27	-0.130	0.451	1.000	36
***SULT2B1***	-0.012	0.946	1.000	37	0.201	0.239	1.000	36	0.198	0.270	1.000	33	0.016	0.937	0.937	27	0.093	0.588	1.000	36
***ESR1***	-0.031	0.845	1.000	42	-0.062	0.700	0.862	41	-0.090	0.592	1.000	38	**-0.364**	**0.048**	**0.384**	**30**	0.020	0.899	1.000	41
***ESR2***	**-0.355**	**0.021**	**0.168**	**42**	-0.087	0.589	1.000	41	-0.070	0.678	1.000	38	0.156	0.411	0.822	30	0.098	0.541	1.000	41
***GPER2***	0.088	0.643	0.935	30	-0.361	0.054	0.864	29	-0.254	0.201	1.000	27	0.201	0.383	0.875	21	0.085	0.660	1.000	29
***GPER34***	0.116	0.540	1.000	30	-0.156	0.420	1.000	29	-0.049	0.810	0.997	27	0.290	0.202	1.000	21	0.048	0.806	0.992	29

Spearman׳s rho correlation coefficient (Rho) and 2-tailed significance (p) and adjusted significance (Adj. p) and N number of endometrial cancer cases are shown.

* Correlation is significant at the 0.05 level (2-tailed).

**Table 3:** Correlations between expression of genes involved in estrogen oxidative metabolism and demographic characteristics of endometrial cancer patients.

**Gene**	**Age**				**Body mass**				**BMI**				**Age at menopause**				**Parity**			
	**Rho**	***p***	**Adj. *p***	***N***	**Rho**	***p***	**Adj. *p***	***N***	**Rho**	***p***	**Adj. *p***	***N***	**Rho**	***p***	**Adj. *p***	***N***	**Rho**	***p***	**Adj. *p***	***N***
***SULT1E1***	0.192	0.255	0.850	37	0.127	0.461	0.922	36	0.122	0.499	0.998	33	-0.213	0.285	0.950	27	0.016	0.925	1.000	36
***CYP1A1***	0.287	0.085	0.850	37	-0.029	0.866	0.962	36	-0.045	0.803	1.000	33	0.003	0.989	0.989	27	-0.086	0.619	0.884	36
***CYP1B1***	0.021	0.900	0.900	37	-0.090	0.602	0.860	36	-0.027	0.881	0.881	33	0.040	0.844	1.000	27	-0.033	0.847	1.000	36
***CYP1A2***	0.238	0.157	0.785	37	-0.144	0.402	1.000	36	-0.119	0.508	0.847	33	0.048	0.811	1.000	27	0.173	0.312	1.000	36
***CYP3A5***	0.028	0.869	0.966	37	-0.130	0.450	1.000	36	-0.155	0.388	1.000	33	0.102	0.612	1.000	27	-0.129	0.453	0.906	36
***CYP3A7***	-0.157	0.353	0.883	37	-0.252	0.139	1.000	36	-0.218	0.222	1.000	33	-0.008	0.970	1.000	27	-0.231	0.175	0.875	36
***COMT***	-0.135	0.426	0.852	37	0.005	0.977	0.977	36	-0.029	0.874	0.971	33	0.209	0.295	0.738	27	-0.288	0.089	0.890	36
***UGT2B7***	0.117	0.490	0.817	37	0.047	0.786	0.983	36	0.032	0.859	1.000	33	0.318	0.106	0.530	27	0.008	0.963	0.963	36
***SULT1A1***	-0.051	0.765	0.956	37	-0.218	0.201	1.000	36	-0.205	0.253	1.000	33	0.059	0.771	1.000	27	-0.167	0.330	0.825	36
***GSTP1***	-0.071	0.684	0.977	35	0.109	0.538	0.897	34	0.140	0.453	1.000	31	0.511^**^	**0.008**	**0.080**	**26**	0.118	0.507	0.845	34

Spearman׳s rho correlation coefficient (Rho) and 2-tailed significance (*p*) and adjusted significance (Adj. *p*) and N number of endometrial cancer cases are shown. ** Correlation is significant at the 0.001 level (2-tailed).

**Table 4:** Correlations between expression of genes involved in progesterone biosynthesis and action and demographic characteristics of endometrial cancer patients.

**Gene**	**Age**				**Body mass**				**BMI**				**Age at menopause**				**Parity**			
	**Rho**	***p***	**Adj. *p***	***N***	**Rho**	***p***	**Adj. *p***	***N***	**Rho**	***p***	**Adj. *p***	***n***	**Rho**	***p***	**Adj. *p***	***N***	**Rho**	***p***	**Adj. *p***	***N***
***PGR***	-0.089	0.563	0.845	45	0.081	0.603	1.000	44	0.016	0.920	0.920	41	-0.076	0.680	0.874	32	0.182	0.238	0.536	44
***PAQR7***	-0.455^*^	**0.002**	**0.018**	**45**	-0.019	0.903	0.903	44	-0.113	0.482	1.000	41	0.277	0.125	0.563	32	0.220	0.150	0.675	44
***PAQR5***	-0.034	0.826	0.826	45	-0.226	0.140	0.630	44	-0.172	0.282	1.000	41	0.049	0.791	0.890	32	-0.124	0.422	0.633	44
***PAQR8***	-0.137	0.382	0.860	43	-0.072	0.653	1.000	42	-0.076	0.646	1.000	39	-0.436^*^	**0.014**	**0.126**	**31**	-0.104	0.514	0.661	42
***PRB***	0.070	0.649	0.834	45	0.072	0.640	1.000	44	0.066	0.683	1.000	41	-0.128	0.485	0.728	32	0.248	0.105	0.945	44
***STAR***	-0.194	0.202	0.606	45	0.358^*^	**0.017**	**0.153**	**44**	0.374^*^	**0.016**	**0.144**	**41**	0.226	0.213	0.479	32	0.207	0.178	0.534	44
***HSD3B1***	0.115	0.491	0.884	38	0.021	0.902	1.000	37	-0.048	0.787	1.000	34	0.008	0.967	0.967	29	0.055	0.748	0.842	37
***HSD3B2***	-0.065	0.700	0.788	37	0.075	0.665	0.998	36	0.044	0.809	0.910	33	-0.194	0.333	0.599	27	-0.014	0.935	0.935	36
***CYP11A1***	-0.243	0.108	0.486	45	-0.050	0.747	0.960	44	-0.139	0.387	1.000	41	0.251	0.166	0.498	32	0.155	0.315	0.567	44

Spearman׳s rho correlation coefficient (Rho) and 2-tailed significance (*p*) and adjusted significance (Adj. *p*) and N number of endometrial cancer cases are shown. * Correlation is significant at the 0.05 level (2-tailed).

**Table 5:** Correlations between expression of genes involved in progesterone metabolism and demographic characteristics of endometrial cancer patients.

**Gene**	**Age**				**Body mass**				**BMI**				**Age at menopause**				**Parity**			
	**Rho**	***p***	**Adj. *p***	***N***	**Rho**	***p***	**Adj. *p***	***N***	**Rho**	***p***	**Adj. *p***	***N***	**Rho**	***p***	**Adj.*p***	***N***	**Rho**	***p***	**Adj.*p***	***N***
***SRD5A1***	-0.212	0.270	0.540	29	-0.531^**^	**0.004**	**0.024**	**28**	-0.533^**^	**0.005**	**0.030**	**26**	-0.218	0.356	0.534	20	-0.382^*^	**0.045**	**0.135**	**28**
***SRD5A2***	0.439^*^	**0.017**	**0.102**	**29**	-0.189	0.336	0.672	28	-0.085	0.678	1.000	26	-0.037	0.876	0.876	20	0.241	0.217	0.326	28
***AKR1C1***	-0.028	0.854	0.854	45	0.010	0.950	1.000	44	-0.048	0.764	0.917	41	0.298	0.097	0.291	32	-0.088	0.568	0.568	44
***AKR1C2***	0.036	0.817	0.980	45	0.003	0.986	0.986	44	-0.080	0.620	1.000	41	0.297	0.099	0.198	32	-0.156	0.313	0.376	44
***AKR1C3***	-0.159	0.459	0.689	24	-0.029	0.895	1.000	23	-0.012	0.960	0.960	21	0.231	0.356	0.427	18	-0.473^*^	**0.022**	**0.132**	**23**
***HSD17B2***	^*^	**0.017**	**0.051**	**45**	0.173	0.262	0.786	44	0.209	0.190	0.570	41	-^*^	**0.043**	**0.258**	**32**	0.245	0.109	0.218	44

Spearman׳s rho correlation coefficient (Rho) and 2-tailed significance (*p*) and adjusted significance (Adj. *p*) and *N* number of endometrial cancer cases are shown. * Correlation is significant at the 0.05 level (2-tailed), **Correlation is significant at the 0.001 level (2-tailed).

**Table 6:** Correlations between expression of genes involved in PGF2α biosynthesis and retinoic acid metabolism and demographic characteristics of endometrial cancer patients.

**Gene**	**Age**				**Body mass**				**BMI**				**Age at menopause**				**Parity**			
	**Rho**	***P***	**Adj. *p***	***N***	**Rho**	***p***	**Adj. *p***	***N***	**Rho**	***p***	**Adj. *p***	***N***	**Rho**	***p***	**Adj. *p***	***N***	**Rho**	***p***	**Adj. *p***	***N***
***AKR1B1***	-0.278	0.064	0.192	45	-0.357^*^	**0.017**	**0.026**	**44**	-0.332^*^	**0.034**	**0.051**	**41**	-0.227	0.211	0.633	32	-0.106	0.495	0.495	44
***AKR1B10***	0.036	0.815	0.815	45	0.481^**^	**0.001**	**0.003**	**44**	0.516^**^	**0.001**	**0.003**	**41**	0.046	0.804	0.804	32	0.249	0.103	0.155	44
***AKR1C3***	-0.159	0.459	0.689	24	-0.029	0.895	0.895	23	-0.012	0.960	0.960	21	0.231	0.356	0.534	18	**-0.473**⁎	**0.022**	**0.066**	**23**

Spearman׳s rho correlation coefficient (Rho) and 2-tailed significance (*p*) and adjusted significance (Adj. *p*) and *N* number of endometrial cancer cases are shown.

⁎ Correlation is significant at the 0.05 level (2-tailed), ​** Correlation is significant at the 0.001 level (2-tailed).

**Table 7:** Changes in expression of genes involved in estrogen biosynthesis and action according to histopathological and clinical characteristics of endometrial cancer patients.

	**Histological grade (high grade*****vs.*****low grade)**		**FIGO stage (IA*****vs.*****IB-IV)**	**Menopausal status**		**Myometrial invasion (yes/no)**	**Myometrial invasion >1/2 (yes/no)**	**Lymphovascular invasion invasion (yes/no)**	
	**Adj.*p***	***p***	***p***	**Adj.*p***	***p***	***p***	***p***	**Adj.*p***	
***AKR1C3***	0.825	0.880	0.306	0.424	0.754	0.555	0.100	0.804	0.99
***CYP19A1***	0.712	0.876	0.219	**0.011**	**0.176**	0.883	0.068	0.680	0.989
***HSD17B2***	**0.010**	**0.160**	0.251	**0.028**	**0.224**	0.939	0.199	0.961	0.961
***HSD17B1***	**0.019**	**0.101**	0.288	0.741	0.847	0.060	0.690	0.075	0.600
***HSD17B4***	**0.010**	**0.080**	0.785	1	1	0.507	0.584	0.364	0.582
***HSD17B8***	0.507	0.676	0.891	0.317	0.845	0.338	0.273	0.117	0.624
***HSD17B14***	0.276	0.552	0.885	0.674	0.899	0.563	0.430	0.359	0.718
***HSD17B12***	0.210	0.480	0.946	0.463	0.741	0.606	0.273	0.215	0.491
***SULT1E1***	0.072	0.230	0.060	0.321	0.734	0.761	0.313	0.177	0.566
***STS***	0.457	0.665	0.915	0.706	0.869	0.580	0.084	0.912	1
***SULT2A1***	0.805	0.920	0.591	0.758	0.809	0.183	0.922	**0.004**	**0.064**
***SULT2B1***	**0.044**	**0.176**	0.737	0.132	0.528	0.493	0.495	0.361	0.642
***ESR1***	0.901	0.901	0.831	0.330	0.66	0.182	0.454	0.210	0.560
***ESR2***	0.333	0.592	0.738	0.113	0.603	0.739	0.255	0.150	0.600
***GPER2***	0.446	0.714	0.799	0.483	0.703	0.919	0.288	0.915	0.976
***GPER34***	0.186	0.496	1	0.268	0.858	0.416	0.507	0.71	0.947

Changes in gene expression ratios were tested using Mann-Whitney nonparametric tests for two-group comparisons, and Wilcoxon nonparametric W tests. Corrections for multiple testing were performed according to Benjamini and Hochberg [7]. Asymptotic 2-tailed significance (*p*) and adjusted significance (Adj. *p*) are shown. Adj. *p* was calculated only in the groups where *p* < 0.05.

**Table 8:** Changes in expression of genes involved in estrogen oxidative metabolism according to histopathological and clinical characteristics of endometrial cancer patients.

	**Histological grade (high grade*****vs.*****low grade)**	**FIGO stage (IA*****vs.*****IB-IV)**	**Menopausal status**	**Myometrial invasion (yes/no)**	**Myometrial invasion >1/2 (yes/no)**	**Lymphovascular invasion invasion (yes/no)**
	***P***	***p***	***p***	***p***	***p***	***p***
***SULT1E1***	0.072	0.060	0.321	0.761	0.313	0.177
***CYP1A1***	0.548	0.737	0.682	0.087	0.126	0.290
***CYP1B1***	0.525	0.568	0.973	0.068	0.143	0.290
***CYP1A2***	0.698	0.591	0.412	0.732	0.313	0.511
***CYP3A5***	0.572	0.402	0.132	0.594	0.255	0.688
***CYP3A7***	0.888	0.481	0.918	0.594	0.626	0.798
***COMT***	0.672	0.840	0.945	0.424	0.097	0.535
***UGT2B7***	0.916	0.149	0.171	0.568	0.922	0.535
***SULT1A1***	0.097	0.920	0.206	0.381	0.922	0.342
***GSTP1***	0.720	0.094	0.806	0.512	0.476	0.147

Changes in expression were tested using Mann-Whitney nonparametric tests for two-group comparisons, and Wilcoxon nonparametric W tests. Corrections for multiple testing were performed according to Benjamini and Hochberg [7]. Asymptotic 2-tailed significance (*p*) is shown.

**Table 9:** Changes in expression of genes involved in progesterone biosynthesis and action according to histopathological and clinical characteristics of endometrial cancer patients.

	**Histological grade (high grade*****vs.*****low grade)**		**FIGO stage (IA*****vs.*****IB-IV)**	**Menopausal status**		**Myometrial invasion (yes/no)**	**Myometrial invasion >1/2 (yes/no)**		**Lymphovascular invasion invasion (yes/no)**
	***p***	**Adj.*p***	***p***	***p***	**Adj.*p***	***p***	***p***	**Adj.*p***	***p***
***PGR***	0.070	0.210	0.337	0.707	1	0.939	0.268	0.402	0.710
***PAQR7***	0.191	0.430	0.165	0.054	0.243	0.417	**0.010**	**0.09**	0.859
***PAQR5***	0.889	1	0.729	0.900	1	0.613	0.180	0.324	0.102
***PAQR8***	0.479	0.719	0.718	0.176	0.528	0.859	0.054	0.122	0.363
***PRB***	**0.026**	**0.117**	0.539	0.802	1	0.530	0.521	0.586	0.616
***STAR***	**0.001**	**0.009**	0.078	0.900	0.9	0.702	**0.041**	**0.123**	0.409
***HSD3B1***	0.904	0.904	0.118	0.693	1	0.252	0.626	0.626	0.224
***HSD3B2***	0.437	0.787	0.202	0.393	0.884	0.704	0.454	0.584	0.742
***CYP11A1***	0.738	0.949	0.157	**0.028**	**0.252**	0.657	**0.036**	**0.162**	0.528

Changes in expression were tested using Mann-Whitney nonparametric tests for two-group comparisons, and Wilcoxon nonparametric W tests. Corrections for multiple testing were performed according to Benjamini and Hochberg [7]. Asymptotic 2-tailed significance (*p*) and adjusted significance (Adj. *p*) are shown. Adj. *p* was calculated only in the groups where *p* < 0.05.

**Table 10:** Changes in expression of genes involved in progesterone metabolism according to histopathological and clinical characteristics of endometrial cancer patients.

	**Histological grade (high grade*****vs.*****low grade)**		**FIGO stage (IA*****vs.*****IB-IV)**	**Menopausal status**		**Myometrial invasion (yes/no)**	**Myometrial invasion >1/2 (yes/no)**		**Lymphovascular invasion invasion (yes/no)**
	***p***	**Adj.*p***	***p***	***p***	**Adj.*p***	***p***	***p***	**Adj.*p***	***p***
***SRD5A1***	0.222	0.444	0.137	0.109	0.218	0.472	0.507	0.507	0.541
***SRD5A2***	0.056	0.168	0.367	**0.048**	**0.144**	0.319	0.352	0.422	0.739
***AKR1C1***	0.486	0.729	0.122	0.920	0.920	0.842	**0.020**	**0.120**	0.987
***AKR1C2***	0.522	0.626	0.088	0.531	0.637	0.842	**0.023**	**0.069**	0.662
***AKR1C3***	0.825	0.825	0.306	0.424	0.636	0.555	0.100	0.200	0.804
***HSD17B2***	**0.010**	**0.060**	0.251	**0.028**	**0.168**	0.939	0.199	0.299	0.961

Changes in expression were tested using Mann-Whitney nonparametric tests for two-group comparisons, and Wilcoxon nonparametric W tests. Corrections for multiple testing were performed according to Benjamini and Hochberg [7]. Asymptotic 2-tailed significance *(p*) and adjusted significance (Adj. *p*) are shown. Adj. *p* was calculated only in the groups where *p* < 0.05.

**Table 11:** Changes in expression of genes involved in PGF2α biosynthesis and retinoic acid metabolism according to histopathological and clinical characteristics of endometrial cancer patients.

	**Histological grade (high grade*****vs.*****low grade)**		**FIGO stage (IA*****vs.*****IB-IV)**	**Menopausal status**		**Myometrial invasion (yes/no)**	**Myometrial invasion >1/2 (yes/no)**	**Lymphovascular invasion invasion (yes/no)**	
	***p***	**Adj.*p***	***p***	***p***	**Adj.*p***	***p***	***p***	***p***	**Adj.*p***
***AKR1B1***	0.070	0.105	0.729	**0.026**	**0.078**	0.842	0.521	0.373	0.56
***AKR1B10***	**0.001**	**0.003**	0.055	0.075	0.113	0.083	0.08	**0.037**	**0.111**
***AKR1C3***	0.825	0.825	0.306	0.424	0.424	0.555	0.1	0.804	0.804

Changes in expression were tested using Mann-Whitney nonparametric tests for two-group comparisons, and Wilcoxon nonparametric W tests. Corrections for multiple testing were performed according to Benjamini and Hochberg [7]. Asymptotic 2-tailed significance (*p*) and adjusted significance (Adj. *p*) are shown. Adj. *p* was calculated only in the groups where *p* < 0.05.

### 1.1 Demographic, histopathological and clinical data

The demographic, histopathological and clinical characteristics are given in Table 1. For the 51 patients, the mean age was 63.16 years (SD, 13.33 years; range, 26.72–83.58 years), the mean body weight was 81.24 kg (SD, 17.25 kg; range, 51–130 kg), and the mean BMI was 30.63 kg/m^2^ (SD, 6.95 kg/m^2^; range, 19.37–48.93 kg/m^2^). According to the WHO definitions, of the 46 patients with BMI data, 10 (21.7%) were within the normal range (BMI, 18.5–25.0 kg/m^2^), 12 (26.1%) were overweight (BMI, 25–30 kg/m^2^), and 24 (52.2%) were obese (BMI, >30 kg/m^2^), with 15 (32.6%) as moderately obese (BMI, 30–35 kg/m^2^), 5 (10.9%) as severely obese (BMI, 35–40 kg/m^2^), and 4 (8.7%) as very severely obese (BMI, >40 kg/m^2^).

Forty-six (92.0%) of the 50 patients with the relevant data had at least one full-term pregnancy, four (8.0%) had none. Information for menopausal status was also available for 50 patients, with 38 (76.0%) post-menopausal and 12 (24.0%) pre-menopausal. The minimum age at the last menstruation was 40 years, and the maximum was 65 years, with the mean of 52.34 years (SD, 4.54 years). The longest post-menopausal time before analysis was 33.6 years.

The Cancer Registry of the Republic of Slovenia was searched for the vital status of these 51 patients. The cut-off point was June 20, 2016, at which date 36 (70.6%) of these patients were still alive, and 15 (29.4%) were dead. The mean age at death was 75.36 years (SD, 9.30 years), with the minimum age at death of 57.05 years, and maximum age of 86.44 years. Eight patients (15.7%) died of cancer: four (7.8%) of uterine cancer, one of ovarian cancer, one of kidney cancer, one of malignant melanoma of the skin, and one of glioblastoma (2.0%, for each). Three patients (5.9%) died of chronic ischemic heart disease, one of atherosclerotic arteries of the extremities, one of benign meningeal neoplasm, and one of infection and inflammatory reaction due to an internal joint prosthesis (2.0%, for each). Morbid obesity with alveolar hypoventilation resulted in the death of one of the patients (2.0%).

The most common histologic type was endometrioid adenocarcinoma, as seen for 41 of the 51 patients (80.4%), of these, 29 (70.7%) had tumor grade 1 (G1), eight (19.5%) grade 2 (G2), and five (12.2%) grade 3 (G3). Five (9.80%) of the 51 patients had serous carcinoma, one (2.0%) had mucinous carcinoma, one (2.0%) had carcinosarcoma, and three (5.9%) had dedifferentiated carcinoma. At histologic examination, the tumor tissue was limited to the endometrium in 12 (23.5%) of the 51 patients, invasion into the myometrium (<50% of myometrial thickness) was seen in 25 (49.0%), and deep tumor invasion (≥50% of myometrial thickness) was seen in 14 (27.5%). For one patient (2.0%), the tumor tissue had spread to the adjacent tissue to the right fallopian tube, while histological examination revealed pelvic lymph node metastases for one patient (2.0%). According to the International Federation of Gynecology and Obstetrics (FIGO) staging, 33 (66.0%) were classified as stage IA, 11 (22.0%) as stage IB, one (2.0%) as stage II, two (4.0%) as stage III, one as stage IIIA, one as stage IIIC1, and one as stage IV (2.0%, for each).

## 2 Experimental design, materials and methods

### 2.1 Gene expression ratios in cancer *versus* adjacent control tissue

We investigated expression of genes encoding enzymes of estrogen biosynthesis [2] (Figure 1, [1]), enzymes of estrogen metabolism [3] (Figure 2, [1]), enzymes of progesterone synthesis and metabolism [4] (Figures 3 and 4, [1]) and enzymes of PGF2α synthesis and metabolism of retinoic acid [5] (Figure 5, [1]). We also investigated expression of nuclear estrogen and progesterone receptors *ESR1*, *ESR2* [2], [6], *PGR*, *PRB* [4]*,* and membrane bound estrogen and progesterone receptors *GPER*, *PAQR5*, *PAQR7* and *PAQR8* (manuscript in preparation) (Figures 2 and 3, [1]). Our studies comprised from 22 to 47 patients. To provide information about up/down-regulation of these 38 genes in cancer *versus* adjacent control tissue we calculated ratios for gene expression in tumor/ adjacent control tissue and these data were statistically analyzed.

### 2.2 Statistical analysis

The correlations between the ratios for expression of 38 genes in tumor/ adjacent control tissue and demographic parameters were evaluated by calculating Spearman׳s correlation coefficient rho (Table 2, Table 3, Table 4, Table 5, Table 6). The statistical significant changes in the ratios for expression of 38 genes in tumor/ adjacent control tissue with regard to histopathological and clinical characteristics were tested using the Mann-Whitney test and the Wilcoxon W test, followed by Benjamini and Hochberg corrections for multiple testing [7] (Table 7, Table 8, Table 9, Table 10, Table 11).
