# Supplementary material for: Data on expression of genes involved in estrogen and progesterone action, inflammation and differentiation according to demographic, histopathological and clinical characteristics of endometrial cancer patients
Source: Data Brief. 2017 May 4;12:632–43. doi: 10.1016/j.dib.2017.04.050 (PMC5430149; doi:10.1016/j.dib.2017.04.050)
Supplement: Supplementary file 1 — Supplementary material [file mmc1.pdf]

Conflict of Interest

We wish to confirm that there are no known conflicts of interest associated with this publication and there has been no financial support for this work that could have influenced its outcome.

We confirm that the manuscript has been read and approved by all named authors and that there are no other persons who satisfied the criteria for authorship but are not listed. We further confirm that the order of authors listed in the manuscript has been approved by all of us.

We confirm that we have given due consideration to the protection of intellectual property associated with this work and that there are no impediments to publication, including the timing of publication, with respect to intellectual property. In so doing we confirm that we have followed the regulations of our institutions concerning intellectual property.

We further confirm that aspect of this work covered in this manuscript that has involved human patients has been conducted with the ethical approval of all relevant bodies and that such approvals are acknowledged within the manuscript.

We understand that the Corresponding Author is the sole contact for the Editorial process. She is responsible for communicating with the other authors about progress, submission of revision and final approval of proofs. We confirm that we have provided a current, correct email address with is accessible by the Corresponding Author.

Signed by all authors as follows:

Maša Sinreih

Saša Štupar

Luka Čemažar

Ivan Verdenik

Snježana Frković Grazio

Špela Smrkolj

Tea Lanišnik Rižner Rižner
